# Neuropharmacological Mechanisms Underlying the Neuroprotective Effects of Methylphenidate

**DOI:** 10.2174/157015908787386041

**Published:** 2008-12

**Authors:** T.J Volz

**Affiliations:** Department of Pharmacology and Toxicology, University of Utah, 30 South 2000 East, Room 201, Salt Lake City, UT 84112, USA

**Keywords:** Dopamine, dopamine transporter, methamphetamine, methylphenidate, neuroprotection, neurotoxicity, Parkinson’s disease, vesicular monoamine transperter-2.

## Abstract

Methylphenidate is a psychostimulant that inhibits the neuronal dopamine transporter. In addition, methylphenidate has the intriguing ability to provide neuroprotection from the neurotoxic effects of methamphetamine and perhaps also Parkinson’s disease; both of which may likely involve the abnormal accumulation of cytoplasmic dopamine inside dopaminergic neurons and the resulting formation of dopamine-associated reactive oxygen species. As delineated in this review, the neuroprotective effects of methylphenidate are due, at least in part, to its ability to attenuate or prevent this abnormal cytoplasmic dopamine accumulation through several possible neuropharmacological mechanisms. These may include 1) direct interactions between methylphenidate and the neuronal dopamine transporter which may attenuate or prevent the entry of methamphetamine into dopaminergic neurons and may also decrease the synthesis of cytoplasmic dopamine through a D2 receptor-mediated signal cascade process, and 2) indirect effects upon the functioning of the vesicular monoamine transporter-2 which may increase vesicular dopamine sequestration through both vesicle trafficking and the kinetic upregulation of the vesicular monoamine transporter-2 protein. Understanding these neuropharmacological mechanisms of methylphenidate neuroprotection may provide important insights into the physiologic regulation of dopaminergic systems as well as the pathophysiology of a variety of disorders involving abnormal dopamine disposition ranging from substance abuse to neurodegenerative diseases such as Parkinson’s disease.

## INTRODUCTION

Methylphenidate (MPD) is a ritalinic acid psychostimulant as shown in Fig. (**1**). It was first synthesized in 1944 [40] and is sold under several brand names including Ritalin and Concerta. MPD is clinically effective in treating both childhood and adult attention-deficit hyperactivity disorder as well as narcolepsy [7, 18, 35, 36, 53, 55]. The use of MPD has also been studied and advocated for weaning patients from mechanical ventilation [23], for treating giggle incontinence [50], and to ameliorate the psychological distress related to both cancer [11, 60] and human immunodeficiency virus infection [12, 76]. 

In the brain, MPD alters the transport of dopamine (DA) across the synaptic plasmalemmal membrane by binding with high affinity to, and thereby competitively inhibiting, the neuronal DA transporter (DAT) [20, 48, 49, 75]. At therapeutic doses of 0.3-0.6 mg/kg, orally administered MPD may actually bind to and occupy more than half of the DAT in the human brain [63]. By inhibiting the DAT, MPD increases extracellular DA concentrations in the brain [55, 61] resulting in a prolonged and/or intensified DA postsynaptic signal. This DAT inhibition and change in the signal timing of DA may, at least in part, mediate the behavioral and locomotor effects of MPD [41, 49]. Additionally, MPD binds to and inhibits the neuronal norepinephrine transporter but has very limited affinity for the serotonin transporter [4, 32]. MPD also binds to both muscarinic and serotonin receptors in the brain [32]. 

MPD has the intriguing ability to protect against methamphetamine (METH) neurotoxicity. This is evidenced by findings that MPD provides complete protection against METH-induced decreases in DA transport when rat striatal synaptosomes are incubated *in vitro* with both METH and MPD [25]. Additionally, *in vivo* MPD post-treatment prevents METH-induced persistent decreases in striatal DA levels, vesicular DA transport, binding of the vesicular mono-amine transporter-2 (VMAT-2) ligand [^3^H]dihydrotetrabenazine (DHTBZ), and vesicular DA content in a rat model of METH neurotoxicity [46]. In dopaminergic neurons, METH is thought to produce this neurotoxicity by causing excess cytoplasmic DA accumulation which, in turn, increases the formation of DA-associated reactive oxygen species that may overwhelm cellular antioxidant systems [3, 8, 13, 17, 66, 69]. 

Abnormal cytoplasmic DA accumulation may also contribute to the development of Parkinson’s disease [5, 22], suggesting the possibility that MPD may be neuroprotective in this disease state as well since MPD treatment has been shown to improve motor function in human Parkinson’s patients [9]. Additionally, MPD provides protection from the behavioral and neurochemical effects of 6-hydroxydopamine in an animal model of Parkinson’s disease [14]. As detailed below, these neuroprotective effects of MPD may be due, at least in part, to its ability to attenuate or prevent abnormal cytoplasmic DA accumulation in dopaminergic neurons by modulating the activity of the DAT and the VMAT-2 through several neuropharmacological mechanisms. 

## MPD NEUROPROTECTION: DIRECT ACTIONS AT THE DAT

**Fig. (1):**
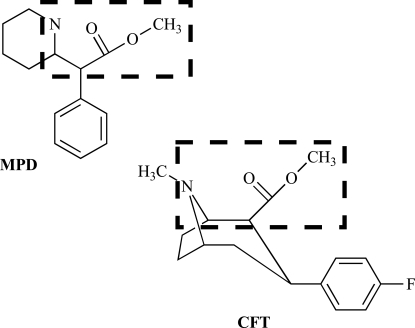
Structures of MPD and the cocaine analog, CFT. The dashed box indicates the superposition of the atomic sequence from the anime nitrogen through the ester group in 3-dimensional space.

The DAT is a plasmalemmal membrane-spanning protein that functions to transport extracellular DA back into the cytoplasm of pre-synaptic dopaminergic neurons. As discussed above, MPD is a DAT inhibitor that competitively binds to a single site on the DAT [48, 49, 75]. MPD shares a DAT binding pharmacophore with cocaine analogs and the sequence of atoms from the amine nitrogen through the ester group of MPD (see Fig. **1**) is superimposed in 3-dimensional space with this same atomic sequence in the cocaine analog, CFT [15]. There are three important types of DAT binding interactions in this shared pharmacophore: a hydrogen bond to the amine nitrogen, one or two hydrogen bonds with the ester group, and hydrophobic binding interactions with the non-polar phenyl ring [6]. The hydrogen bonding interactions with the ester group involve arginine residues on the DAT [64, 70, 71]. The phenyl ring fits into a hydrophobic binding pocket on the DAT made up of several amino acid residues. The hydrogen bonding interactions are very specific whereas the hydrophobic binding pocket can accept a variety of atomic geometries among various DAT inhibitors. The phenyl rings of MPD and the cocaine analogs may thus fit into the same hydrophobic pocket on the DAT despite their differences in molecular orientation. This is supported by findings that MPD, cocaine, and cocaine analogs do indeed share the same binding site on the DAT [48, 54, 75]. 

Through these direct binding interactions with the DAT, MPD may exert its neuroprotective effects through at least two possible neuropharmacological mechanisms. In the first possible mechanism, MPD may inhibit the DAT and thereby possibly attenuate or prevent METH from gaining entry into the neuron and causing DA to efflux from the vesicles. This, in turn, may prevent the METH-induced accumulation of excess cytoplasmic DA and the resulting formation of DA-associated reactive oxygen species. In support of this possible mechanism, DAT-knockout mice are resistant to the neurotoxicity produced by multiple METH administrations [16]. Additionally, the DAT inhibitors amfonelic acid, mazindol, bupropion, and benztropine block or attenuate the neurotoxic effects of METH in rat striatum [31]. These authors concluded that “an intact and functional DA uptake site is necessary for the development of METH-induced long-term DA depletions.” MPD can also prevent the neurotoxic effects of 1-methyl-4-phenylpyridinium (MPP^+^) in both human embryonic kidney 293 cells expressing the human DAT and in rat embryonic mesencephalic cultures by blocking the DAT [30]. 

In order for this proposed neuropharmacological mechanism of MPD neuroprotection to be valid, METH must be transported by the DAT and not diffuse passively across the plasmalemmal membrane. Evidence supporting this assumption can be obtained from empirical calculation. METH has a pK_a_ of 10.1 [1, 42] and, depending upon the pH, can exist in either a neutral uncharged form or a cationic form as shown in Fig. (**2**). The relative abundance of neutral and cationic forms is governed by the Henderson-Hasselbalch equation of pH = pK_a_ + log ([neutral]/[cation]). Using this equation and the pK_a_ of METH, approximately 0.2% of METH exists in the neutral uncharged form at a physiological pH of 7.4. The neutral uncharged form of METH is lipophilic with a log *P* value of 2.10 while the cationic form is very unlipophilic with a log *P* value of -2.03 [19]. Thus, at physiological pH it is possible that only the fraction of METH which exists in the neutral uncharged form can passively diffuse across the plasmalemmal membrane while the METH which exists in the cationic form requires the DAT for transport. This same empirical calculation approach has also recently been used to calculate the abundance of amphetamine species that can diffuse across vesicular membranes [73]. 

Experimental evidence also suggests that METH and other amphetamines may be transported by the DAT. For example, amphetamine accumulation into striatal synaptosomes is saturable, temperature-dependent, and inhibited by MPD [78]. METH elicits "DA-like" transporter-associated currents in Xenopus oocytes expressing the human DAT [52]. Likewise, the transport of amphetamine into human embryonic kidney 293 cells expressing the human DAT is Na^+^-dependent and produces an inward current [51]. DAT-mediated accumulation of METH into cultured neuronal cells expressing the DAT is directly correlated with temperature [77]. Finally, amphetamine competes with DA for a common binding site on the striatal DAT [74]. Thus, both empirical calculation and experimental evidence may support the assumption that METH, at least at low concentrations, may be actively transported by the DAT into dopaminergic neurons which may support the first possible mechanism of MPD neuroprotection. 

However, it should be noted that at higher METH concentrations there may be a sufficient quantity of the neutral uncharged form of METH to diffuse across the plasmalemmal membrane and exert biological effects even though the neutral uncharged form is not the predominant species in terms of relative abundance. For example, 10 μM METH can enter nerve terminals and deplete vesicular DA stores in brain slices prepared from mice lacking the DAT [24]. Additionally, Liang and Rutledge [28] proposed a model of amphetamine-induced neuronal DA release in which low doses of amphetamine are transported by the DAT while amphetamine diffuses across the plasmalemmal membrane at higher doses. 

MPD may also provide neuroprotection *via* a second possible neuropharmacological mechanism involving direct interactions with the DAT. In this possible mechanism, MPD binding to the DAT may inhibit the transport of DA into dopaminergic neurons. This may increase extracellular DA concentrations which, in turn, may increase D2 receptor activation. Increased D2 receptor activation may then decrease tyrosine hydroxylase activity and thereby decreases cytoplasmic DA synthesis. The end result of this D2 receptor-mediated cascade of events may be a reduction in both the accumulation of cytoplasmic DA and the production of DA-associated reactive oxygen species. In support of this second possible neuropharmacological mechanism, D2 receptor activation has been shown to inhibit tyrosine hydroxylase activity and DA synthesis in a mouse mesencephalic cell line [39] and to also inhibit DA synthesis in striatal synaptosomes [57]. Additionally, *in vivo* MPD administration has been shown to decrease DA synthesis in rats [38]. However, it should be noted that D2 receptor activation may also increases DAT-mediated transport of DA into the cytoplasm by increasing DAT cell surface expression [33, 34] which may act to attenuate the possible neuroprotective effects of this proposed mechanism. 

## MPD NEUROPROTECTION: INDIRECT ACTIONS ON THE VMAT-2

The VMAT-2 is a vesicular membrane-spanning protein that functions to transport the cytoplasmic DA inside of neurons into vesicles for storage and subsequent release. The VMAT-2 is thus an important regulator of both cytoplasmic DA levels and dopaminergic function. Neuronal VMAT-2-containing vesicles may be classified as either cytoplasmic vesicles (i.e., those vesicles that *do not* co-fractionate with synaptosomal membranes after the osmotic lysis of striatal synaptosomes) or membrane-associated vesicles (i.e., those vesicles that *do* co-fractionate with synaptosomal membranes after osmotic lysis) [65]. DA transport into cytoplasmic vesicles follows Michaelis-Menten kinetics while DA transport into membrane-associated vesicles has large positive substrate cooperativity (i.e., kinetics that are different from Michaelis-Menten kinetics) [65, 68]. The membrane-associated vesicles also have a large DA sequestration capacity and may therefore function as a reserve sequestration capacity or “DA sink” to prevent cytoplasmic DA from rising to aberrant levels [65]. 

Synaptic vesicles may coexist in differing functional states or pools. These include the readily releasable vesicles that are docked at the plasma membrane awaiting exocytosis, the recycling vesicles that are undergoing reformation and recycling following exocytosis, and the reserve vesicles that are not immediately involved in exocytosis and are at a more distant location further away from the membrane [44, 45]. It has been hypothesized that “synaptic vesicles released by osmotic lysis from isolated synaptosomes presumably largely correspond to the reserve pool” [37] and thus the cytoplasmic vesicles may correspond to the reserve vesicles. Additionally, “vesicles remaining associated with lysed synaptosomes may be associated with the presynaptic cytomatrix or are docked to the plasma membrane and thus belong to the recycling and/or readily releasable pool” [37]. The membrane-associated vesicles may thus correspond to the recycling/ readily releasable vesicles, especially since the membrane-associated vesicle subcellular fraction contains the readily releasable vesicle/active zone marker piccolo [65]. 

The disruption of VMAT-2 function in vesicles likely contributes to neurotoxicity, since heterozygous VMAT-2 knock-out mice have increased METH-induced neurotoxicity [17]. Additionally, pretreatment with the VMAT-2 inhibitor, reserpine, potentiates METH-induced neurotoxicity [56, 72]. Finally, METH-induced accumulation of oxygen radicals and damage to DA neurites varies inversely with VMAT-2 expression levels in postnatal ventral midbrain neuronal cultures [27]. 

Multiple high-dose administrations of METH decrease vesicular DA transport in cytoplasmic vesicles purified from rat striata [2]. METH treatment also decreases both DA transport and binding of the VMAT-2 ligand, [^3^H]DHTBZ, in cytoplasmic vesicles purified from mice striata [21]. However, there is no significant loss of [^3^H]DHTBZ binding in whole striatal homogenates prepared from treated mice. These and other vesicle trafficking data suggest that multiple high-dose METH administrations cause a subcellular redistribution of VMAT-2 containing vesicle out of the cytoplasm such that fewer VMAT-2, and presumably associated cytoplasmic vesicles, are available to sequester DA [43, 46, 59]. 

MPD and lobeline post-treatments that reverse these METH-induced alterations in VMAT-2 redistribution or function also prevent neurotoxicity after multiple high-dose METH administrations [10, 46]. The neuroprotective effect of lobeline is partly due to the attenuation of METH-induced hyperthermia, although lobeline may also attenuate glutamate-induced exitotoxicity after METH administration [10]. MPD may exert its neuroprotective effects through at least two different possible neuropharmacological mechanisms involving indirect actions (as opposed to direct binding interactions with the DAT as discussed above) on the DA sequestration function of the VMAT-2. The first possible neuropharmacological mechanism involves the cytoplasmic vesicles while the second possible mechanism involves the membrane-associated vesicles. In the first mechanism, MPD may redistribute VMAT-2-containing vesicles within striatal dopaminergic nerve terminals away from membranes and into the cytoplasm which may result in the movement of vesicles to a subcellular region left deficient of VMAT-2 activity because of the actions of METH [46, 47]. MPD may thus increase vesicular DA sequestration in that region and perhaps compensate for any METH-induced cytoplasmic DA accumulation. In this way, MPD may reverse or prevent degenerative processes associated with decreased VMAT-2 function and aberrant DA storage. 

This possible mechanism of neuroprotection is supported by the experimental findings that MPD increases and decreases VMAT-2 immunoreactivity in the cytoplasmic and membrane-associated vesicle subcellular fractions, respectively, without altering total synaptosomal VMAT-2 content [47]. Binding of [^3^H]DHTBZ is affected similarly [47]. The trafficking of vesicles away from membranes and into the cytoplasm increases DA transport into cytoplasmic vesicles by simply increasing the number of VMAT-2 containing cytoplasmic vesicles (as opposed to altering the function of each individual VMAT-2 protein) [46, 47, 65]. MPD thus increases DA transport by increasing both the V_max_ of DA transport and the density of kinetically active VMAT-2 without changing the K_m_, the catalytic rate constant, or the rate constant for DA binding to the VMAT-2 [65]. This suggests that the kinetics of DA binding to the VMAT-2 and the translocation of DA across the vesicular membrane are unaltered [65, 67]. MPD also increases the DA content of purified vesicles without altering total tissue DA content [46, 65] which suggests that MPD redistributes DA within the nerve terminals, presumably as a consequence of the redistribution of vesicles. These MPD-induced vesicle trafficking effects are D2 receptor-mediated as the D2 receptor antagonist, eticlopride, attenuates or blocks these effects and the D2 receptor agonist, quinpirole, mimics the effects of MPD on cytoplasmic vesicles [47, 58]. This suggests that these trafficking effects are likely due to the ability of MPD to increase extracellular DA concentrations by blocking the DAT [26, 62]. 

In the second possible neuropharmacological mechanism of neuroprotection, MPD actually alters the functioning of VMAT-2 in the membrane-associated vesicles to transport more DA. This mechanism is supported by the findings that, in addition to redistributing vesicles away from membranes and into the cytoplasm, MPD also increases vesicular DA sequestration into the remaining membrane-associated vesicles [65]. MPD thus kinetically upregulates the VMAT-2 protein itself in the remaining vesicles such that a larger quantity of DA is transported [65]. By increasing membrane-associated vesicular DA sequestration in this manner, MPD may attenuate or prevent the degenerative processes associated with METH-induced decreased VMAT-2 function and cytoplasmic DA accumulation. Because abnormal cytoplasmic DA accumulation also likely contributes to the development of Parkinson’s disease [22], the potential of MPD-induced increases in vesicular DA sequestration (caused by vesicle trafficking in the cytoplasmic vesicles and by kinetic upregulation of VMAT-2 in the membrane-associated vesicles) to attenuate the disease’s progression merits further investigation; as suggested by findings that MPD treatment improves motor function in Parkinson’s patients [9]. 

These MPD-induced increases in vesicular DA sequestration may have several additional interesting functional consequences. The increase in vesicular DA transport increases vesicular DA content with no change in whole striatal tissue DA content [46, 65]. By increasing vesicular DA transport velocities, MPD thus redistributes DA within the striatum from the cytoplasm and into the vesicles. As a consequence of increased vesicular DA sequestration and DA content, MPD also increases the speed and extent of stimulated DA release from striatal suspensions [65]. The amount of vesicular DA content and the speed of neurotransmitter release can influence receptor activation [29], and MPD may thus influence quantal synaptic transmission in the striatum by increasing the rate at which DA receptors are exposed to DA, and perhaps the magnitude and/or duration of this effect. 

## CONCLUSION

The studies reviewed above help us to understand how MPD has the ability to provide neuroprotection against METH-induced neurotoxicity and perhaps Parkinson’s disease through possible mechanisms involving direct interactions with the DAT and additional mechanisms involving indirect effects upon the VMAT-2. These mechanisms, summarized in Fig. (**3**), attenuate or prevent the abnormal accumulation of cytoplasmic DA and the resulting formation of potentially neurotoxic DA-associated reactive species. At present, it is difficult to quantify the amount that any individual mechanism contributes to MPD neuroprotection *in vivo*. Further studies investigating the neuropharmacology, biochemistry, and molecular biology underlying these mechanisms are warranted as these will help to determine the *in vivo* contribution of each mechanism. These additional studies may also provide insight into the physiologic regulation of dopaminergic systems as well as the pathophysiology of a variety disorders involving abnormal DA disposition ranging from substance abuse to neurodegenerative diseases such as Parkinson’s disease. Ultimately, this may suggest leads for developing related novel and more effective neuropharmacologic therapeutic treatment strategies.

## Figures and Tables

**Fig. (2):**
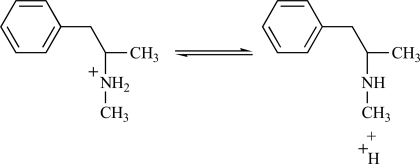
Because the amine nitrogen of METH can accept or donate a proton, METH exists in an equilibrium between a neutral uncharged form (shown right) and a positively charged cationic form (shown left).

**Fig. (3):**
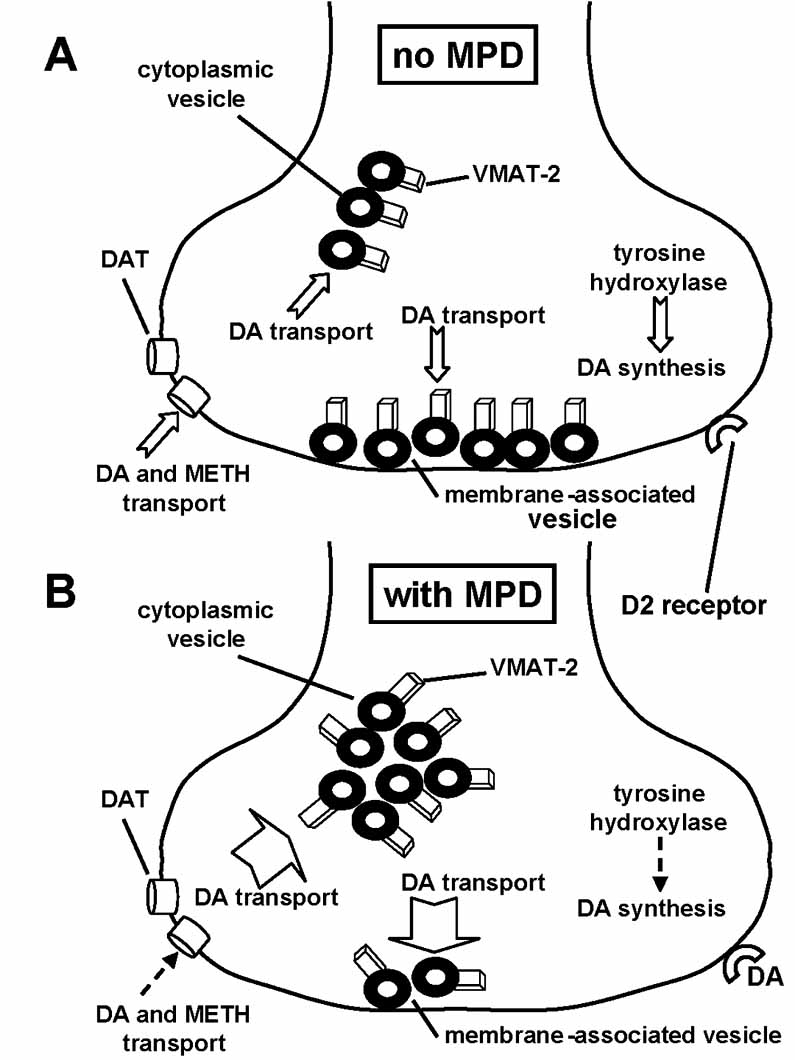
The neuroprotective effects of MPD are caused by at least four possible mechanisms: two involving direct interactions with the DAT and two involving indirect effects upon the VMAT-2. These mechanisms, shown in Panel A (no MPD) and Panel B (with MPD), may decrease DAT-mediated METH transport and cytoplasmic DA synthesis while also increasing DA transport into cytoplasmic and membrane-associated vesicles.
